# Identifying individual-specific microbial DNA fingerprints from skin microbiomes

**DOI:** 10.3389/fmicb.2022.960043

**Published:** 2022-10-06

**Authors:** Yiluan Zheng, Jianlu Shi, Qi Chen, Chao Deng, Fan Yang, Ying Wang

**Affiliations:** ^1^Department of Automation, Xiamen University, Xiamen, China; ^2^National Institute for Data Science in Health and Medicine, Xiamen University, Xiamen, China; ^3^Stomatological Hospital of Xiamen Medical College, Xiamen, China; ^4^Xiamen Key Laboratory of Stomatological Disease Diagnosis and Treatment, Xiamen, China; ^5^Xiamen Key Laboratory of Big Data Intelligent Analysis and Decision, Xiamen, China; ^6^Fujian Key Laboratory of Genetics and Breeding of Marine Organisms, Xiamen, China

**Keywords:** skin microbiome, metagenomics, human identification, microbial DNA fingerprint, individual-specific *k*-mer

## Abstract

Skin is an important ecosystem that links the human body and the external environment. Previous studies have shown that the skin microbial community could remain stable, even after long-term exposure to the external environment. In this study, we explore two questions: Do there exist strains or genetic variants in skin microorganisms that are individual-specific, temporally stable, and body site-independent? And if so, whether such microorganismal genetic variants could be used as markers, called “fingerprints” in our study, to identify donors? We proposed a framework to capture individual-specific DNA microbial fingerprints from skin metagenomic sequencing data. The fingerprints are identified on the frequency of 31-mers free from reference genomes and sequence alignments. The 616 metagenomic samples from 17 skin sites at 3-time points from 12 healthy individuals from Integrative Human Microbiome Project were adopted. Ultimately, one contig for each individual is assembled as a fingerprint. And results showed that 89.78% of the skin samples despite body sites could identify their donors correctly. It is observed that 10 out of 12 individual-specific fingerprints could be aligned to *Cutibacterium acnes*. Our study proves that the identified fingerprints are temporally stable, body site-independent, and individual-specific, and can identify their donors with enough accuracy. The source code of the genetic identification framework is freely available at https://github.com/Ying-Lab/skin_fingerprint.

## Introduction

The skin is the largest organ of the human body. It is a critical ecosystem between the human body and the external environment, harboring microbial communities in various physiologically and topographically distinct niches (Grice et al., 2009). Metagenomic shotgun sequencing provides high resolution to characterize the microbial community at the nucleotide level. Systematic metagenomic investigations of the human skin microbiome were initiated and further expanded in the Human Microbiome Project (HMP) and integrative HMP (iHMP), respectively, by large-scale multi-site (Oh et al., 2014) and temporal (Oh et al., 2016) sequencing data. The metagenomic samples produced across 17 skin sites at 3-time points for nearly 3 years from 12 healthy individuals made it possible to study the full-scale effects of biogeographic, individual, and temporal factors. Based on reference sequences, the rational analysis of the composition and function of the healthy skin microbiome was conducted on bacterial, fungal, and viral communities. Furthermore, studies have demonstrated that biogeography and individuality significantly shape a community's functional and taxonomic characteristics (Oh et al., 2014) and that skin microbiomes were largely stable over time, despite their exposure to the external environment (Oh et al., 2016).

Previous studies have shown that the similarity of microbiota within an individual is more significant than that between individuals (Oh et al., 2014, 2016; Schmedes et al., 2017; Woerner et al., 2019). Microbial abundance profiles in feces, skin, saliva, soil, and plant materials can potentially link individuals to criminal activities, offering another complement to existing forensic identification technologies (Neckovic et al., 2020). Previous studies showed that microorganisms on human skin and gut are very similar at the phylum level, while the diversity of each individual is very specific at the level of genera, species, and strains of specific populations (Krishna, 2018). Therefore, it is worthwhile to study the differences in skin microbiomes among individuals and whether such differences are significant enough to identify a specific individual, especially in a forensic context. Studies performed by Ol et al. showed that even highly variable individual skin data from long-term exposure to the external environment remained in a relatively stable state, making it possible to use microbial communities within the skin microbiome to identify individuals (Oh et al., 2014, 2016). Also, a specific person could even be identified through microbes deposited on the surface of cell phones or computer mice (Tozzo et al., 2020).

Furthermore, the systematic studies of Budowle's group demonstrate the existence of microbial fingerprints in skin microbiomes (Schmedes et al., 2017, 2018), especially focusing on identifying a particular individual from several candidates based on the samples from the skin at the identical body site. Their experiments confirmed that core skin microbial species are stable over time and shared by all individuals as microbial signatures of a fixed body site for each individual (Schmedes et al., 2017). They found that the individual identifying accuracy of clade-specific markers ranged from 56.67 to 100% with a mean accuracy of 82.20%. Furthermore, the Budowle group developed a targeted sequencing method called hidSkinPlex (Schmedes et al., 2018), including 286 bacterial (and phage) for individual identification. Throughout their studies, the Budowle group focused on identifying individual skin microorganisms on the same body site from different individuals.

However, previous explorations (Fierer et al., 2010; Oh et al., 2014, 2016; Schmedes et al., 2017, 2018; Woerner et al., 2019; Tozzo et al., 2020) verified that microorganisms in each individual's skin microbiome have specific genetic variants. Moreover, the fact that various body sites can come into frequent contact leads to the exchange of microorganisms (Lloyd-Price et al., 2017). This suggests, in turn, that an individual's whole-body skin microbiome might consist of a common set of genetic variants, or skin microbial DNA markers, thus making it possible to use any body site, instead of just specific sites, to identify a particular person. Therefore, in this study, we asked the following two questions: Do there exist strains or genetic variants in skin microorganisms that are individual-specific, temporally stable, and body site-independent? And if so, whether such microorganismal genetic variants could be used as fingerprints to identify donors. Accordingly, we proposed a framework to capture individual-specific DNA microbial fingerprints. Free from reference genomic sequences and sequence alignments, the long *k*-mer (*k* = 31) spectrum-based model from our previous studies (Wang et al., 2018, 2020) is adopted to identify genetic variants present in the whole-body skin of one individual, but absent from all body sites of other individuals based on metagenomic sequencing data. We termed this individual-specific fingerprinting. The individual-specific genetic variants are identified and represented by individual-specific *k*-mers, filtered, and assembled into individual-specific contigs as individual-specific DNA microbial fingerprints. We adopted skin data of 12 healthy individuals produced by iHMP, including metagenomic sequencing samples from 17 skin sites at 3-time points for nearly 3 years. In total, we identified 18,0321 individual-specific 31-mers and assembled them into 65,648 individual-specific contigs for the 12 individuals, with the length from 34 to 29,996 bp. Filtered by abundance-difference significance testing, overlapping, and length of contigs, we kept one contig as skin DNA microbial fingerprints for each individual.

To obtain more biological information about genetic variants, the identified individual-specific contigs were aligned to the genomic reference sequences of microorganisms in NCBI (Coordinators, 2016) by BLAST (Altschul et al., 1990). We found that 10 out of 12 individual-specific fingerprints could be aligned to *Cutibacterium acnes* (previous name *Propionibacterium acnes*), which is consistent with the observation that *C. acnes* from the skin is the greatest temporal stable (up to almost 3 years) in single-nucleotide variant (SNV) profiles as individual-specific microbiome features (Oh et al., 2016). Furthermore, instead of designing complicated classification models, we only used the abundance (RPKM, Reads Per Kilobase per Million mapped reads) threshold of fingerprints as a decision rule to separate individuals. By only using fingerprint abundance as decision variable, a decision tree was designed with the ability to identify 7 out of 12 individuals with almost 100% accuracy and total accuracy of 89.78%.

## Materials and methods

### The framework for detecting individual-specific microbial DNA fingerprints

**Figure 1:**
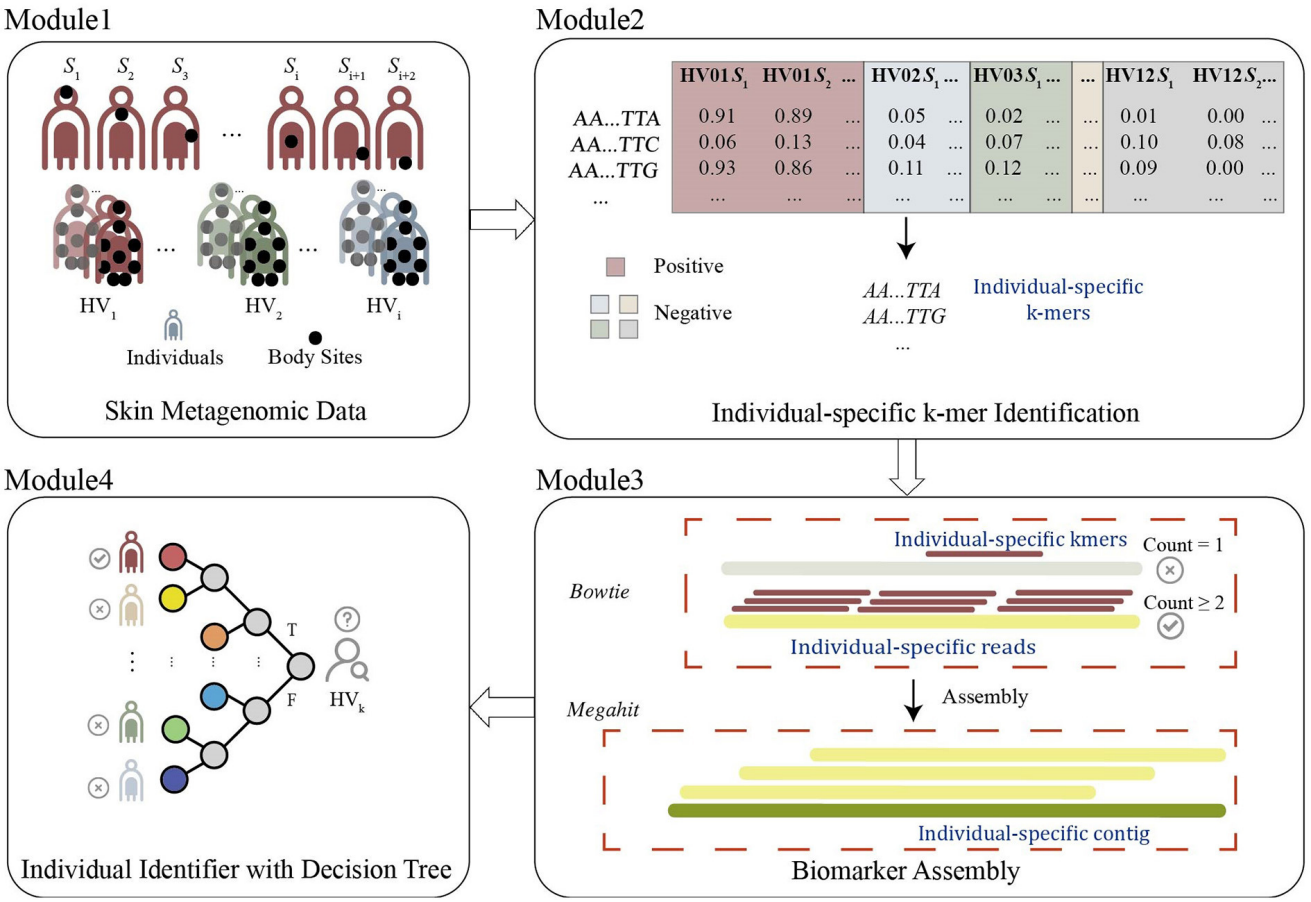
The framework to detect individual-specific microbial DNA fingerprints includes four modules: Module 1-data preparation removes outliner samples, and divides the dataset into balanced training and testing sets. Module 2- individual-specific *k*-mers detection identifies *k*-mers with significantly present/absent or abundant difference. Module 3- individual-specific markers production assembles the individual-specific *k*-mers to contigs as individual-specific microbial DNA marks. Module 4- an individual-identifying decision tree identifies individuals with individual-specific markers.

The framework to detect individual-specific microbial DNA fingerprints is shown in Figure 1. The process is composed of four modules. (1) Data preparations: Metagenomic sequencing data from different body sites and different individuals are pre-processed to filter out the data with extremely low sequencing depth, to avoid incomplete coverage of the metagenome, and to remove samples from body sites strongly related to living habits and affected by the living environment. The remained data are divided into balanced training and testing sets. (2) Detecting individual-specific *k*-mers: All samples from a certain individual are labeled as positive, and the others are negative. The individual-specific *k*-mers detected in cross-validation of the training process are evaluated one by one on the testing data. The *k*-mers passing the test are listed as individual-specific *k*-mers. The procedure is repeated to detect each individual's specific *k*-mers. (3) Producing individual-specific markers: For each individual, his/her specific *k*-mers are aligned to the original sequencing reads, and reads perfectly matched by more than two specific *k*-mers are collected. These reads are then assembled into contigs, and the contigs which have a significant difference in abundance by RPKM between the current individual and others are specified as individual-specific contigs. It is the individual-specific contig that is adopted as the individual's microbial marker. The procedure is repeated until all individuals obtain their specific markers. (4) Designing individual identification with a decision tree: For each individual, the RPKM threshold of the individual-specific contig is used as the DNA microbial fingerprint to separate individuals. The markers for all individuals are finally combined to build a compound logic individual discriminant by a decision tree, and each individual can be identified as a leaf of the decision tree.

Our framework processes skin metagenomic sequencing data, which were collected from multiple body sites of different individuals. Therefore, for each individual, there are multiple metagenomic samples.

#### Module 1: Data preparation

Data preparation is performed to achieve two goals: (1) select and preprocess metagenomic sequencing data with as much information reserved as possible and (2) introduce undersampling for correctly handling a highly unbalanced dataset to avoid bias.

The sequencing data with extremely low sequencing depth were removed to avoid highly-incomplete coverage of the metagenome. In our study, the samples whose read number is smaller than 1/10 of the median number of all samples are considered as extremely low sequencing depth and removed. And the samples from body sites strongly related to living habits and affected by the living environment were also removed from the dataset.

The remained data are divided into balanced training and testing sets. Samples from one specific individual are labeled as positive, and the others are negative, followed by randomly dividing into a ratio of 70% training dataset and 30% testing dataset. Since the number of negative samples is more than ten times the number of positive samples, it is essential to take measures to avoid the impact of data imbalance on subsequent work. A more appropriate approach to deal with large unbalanced datasets is undersampling. For the training set in our study, positive data were randomly sampled from all positive data, and negative data were randomly undersampling selected from the data of the other individuals.

#### Module 2: Detecting individual-specific *k*-mers

The “*k*-mer” means k-bp nucleotide string in this study, and the total number of all possible *k*-mers is 4^*k*^. The “individual-specific *k*-mer” means *k*-mer is present in most of one individual's body skin sites, but absent from all body sites of other individuals. Hence, those *k*-mers are unique to that one individual. Individual-specific *k*-mers are the smallest element used to identify individuals. By using *k*-mers as the basic element, the framework is free from reference sequence and sequence alignment. The detection of individual-specific *k*-mers on sequencing data involves three steps.

##### *k*-mer counting and feature logicalization

For training data, KMC3 (Kokot et al., 2017) is adopted to calculate the occurrence of *k*-mers in each sequencing datum, and only *k*-mers that appear more than twice are kept. The reverse complements of reads were taken into consideration. A vector of *k*-mer counting is generated for each sequencing sample.

For the selection of *k*-mer length, the previous study showed that sufficiently long *k*-mers are usually specific to a genome (Fofanov et al., 2004). When *k* ≥ 30, the average ratio of common *k*-mers between the genomes is less than 1.02% on 100 pairs of bacterial genomes (Vinh et al., 2015). However, a longer *k*-mer requires a large sample size to guarantee high specificity, the huge computing memory and storage. According to a previous study, the *k*-mer with a length 30–40 is a reasonable trade-off among sensitivity, specificity, and computational cost (Wang et al., 2018). Therefore, in our study, we chose *k* = 31.

The *k*-mer frequency vector is represented as a logicalized feature vector with 1/0 to represent *k*-mers as present or absent in one sample, as shown in Equation (1),


(1) $$\begin{array}{c} f_{i}^{\left(l\right)}\left(j\right)=\{\begin{array}{c} 1if\;f_{i}\left(j\right)>0\\ 0if\;f_{i}\left(j\right)=0 \end{array}\; \end{array}\;$$

where *f*_*i*_(*j*) is the counting of occurrence of *k*-mer *i* in the sequences data of sample *j*. $f_{i}^{\left(l\right)}\left(j\right)$ is the logical value of *k*-mer *i* in sample *j*, and the superscript *l* indicates the logical feature.

##### *k*-mer matrix over training samples

The vectors of *k*-mer counts in each sequencing sample are normalized by the sum of the number of *k*-mers that occur in the sample, denoted as *n*_*i*_(*j*). The *k*-mer vectors of two groups of training samples are merged into a *k*-mer frequency union matrix, where each row represents a *k*-mer, and each column represents a sequencing sample, as shown in Equation (2), where *f*_*m*_(*n*) indicates the frequency of *k*−*mer*_*m*_ in the *sample*_*n*_.


(2) $$\begin{array}{c} \begin{array}{c} k-mer_{1}\\ k-mer_{2}\\ \begin{array}{c} \;\vdots \\ k-mer_{m} \end{array} \end{array}\left[\begin{array}{c} \begin{array}{c} \;Positive\;S1\;PositiveS2\;\cdots \end{array}\begin{array}{c} \;Negative\;S1Negative\;S2\;\cdots \end{array}\\ \begin{array}{c} \;n_{1}\left(1\right)\;n_{1}\left(2\right)\cdots \\ \;n_{2}\left(1\right)\;n_{2}\left(2\right)\cdots \\ \begin{array}{c} \;\vdots \\ \;n_{m}\left(1\right) \end{array}\begin{array}{c} \;\vdots \\ \;n_{m}\left(2\right) \end{array}\begin{array}{c} \;\vdots \\ \cdots \end{array} \end{array}\begin{array}{c} \begin{array}{c} \;n_{1}\left(n\right)\;n_{1}\left(\;n+1\right)\;\cdots \end{array}\\ \begin{array}{c} \;n_{2}\left(n\right)n_{2}\left(n+1\right)\;\cdots \end{array}\\ \begin{array}{c} \begin{array}{c} \;\vdots \\ \;n_{m}\left(n\right) \end{array}\begin{array}{c} \;\vdots \\ \;n_{m}\left(n+1\right) \end{array}\begin{array}{c} \;\vdots \\ \;\cdots \end{array} \end{array} \end{array} \end{array}\right] \end{array}\;$$

The “highly-sparse” feature means that a *k*-mer does not exist in most training samples, i.e., the frequencies of a *k*-mer are 0 in most training cases and most training controls. Then those features have very limited contributions to classification. Therefore, if a *k-*mer is absent in more than 80% of negative samples and 80% of positive samples, the *k*-mer is removed from the feature set. The stringent threshold of 80% offers high confidence in filtering out less useful features.

##### Detecting individual-specific *k*-mers

For the remained *k*-mers, we use each *k*-mer to design a logical predictor to evaluate the discriminant ability of the current *k*-mer. The logical predictor is designed as Equation (3), which predicts negative or positive based on whether a *k*-mer *I* is present in the sequencing data of sample *j* or not.


(3) $$y\left(j\right)=\{\begin{array}{c} 1if\;f_{i}^{\left(l\right)}\left(j\right)=1\\ 0if\;f_{i}^{\left(l\right)}\left(j\right)=0 \end{array}$$

or


$$y\left(j\right)=\{\begin{array}{c} 1if\;f_{i}^{\left(l\right)}\left(j\right)=0\\ 0if\;f_{i}^{\left(l\right)}\left(j\right)=1 \end{array}$$

where *y*(*j*) = 1/0 is the logical predictor that sample *j* belongs to Group+/Group-, The prediction performance of the current *k*-mer is evaluated by ASS, an average of sensitivity and specificity, defined as Equation (4).


(4) $$\begin{array}{l} ASS=\frac{\left(Sensitivity+Specificity\right)}{2} \end{array}$$

If a *k*-mer achieves *ASS* ≥ θ_1_, the corresponding *k*-mer is identified to be individual-specific. The individual-specific *k*-mers are present in one individual but absent in others. In our study, θ_1_ is set as 0.80, which means that the current *k*-mer alone can separate the positive individual from the other negative individuals on the training samples with ASS ≥ 0.8.

The identification of individual-specific *k*-mers is implemented by KmerGO, a user-friendly tool to identify the group-specific sequences on two groups of high-throughput sequencing datasets, see Wang et al. (2018, 2020) for details.

#### Module 3: Producing individual-specific markers

Individual-specific *k*-mers were aligned to the individual's sequencing reads by Bowtie (Langmead, 2010) with mismatch = 0. Reads perfectly matched by more than two *k*-mers were collected and assembled into individual-specific contigs by MEGAHIT (Li et al., 2015) with default running mode and parameters. The assembled contigs within an individual have many overlaps owing to the assembly strategy. The reason for using assembled contigs instead of 31-mers are: (1) For the 12 candidates, there are 10^3^-10^5^ individual-specific 31-mers identified, which means that the fingerprints might be much longer than 31bp. And the longer the fingerprint is, the more unique the fingerprint would be in a larger population. (2) For each individual, there are lots of 31 mers with the highest ASS, it is hard to decide which one to select. And as a biomarker, there should be a sequence with enough length. Moreover, the *k*-mer with the highest ASS does not necessarily have enough generalization. (3) From the observation of the identified *k*-mers, one 31 mer might only contain 1 genetic variant. And in the assembled sequences, there exist two genetic variants. One example is shown in HV12 in Figure 8, which means that the longer the fingerprint is, the more genetic variant information is contained.

First, we only kept the contigs with sufficient abundance, in our study, of greater than 0.01 RPKM.

Second, among the remained contigs, we use the classification performance of each contig on the training set to evaluate its ability to separate the corresponding individual from others and determine one contig as the fingerprint marker of the current individual. In detail, for each contig of each individual, we attempted to find an abundance RPKM threshold of the fingerprint to discriminate one particular individual from others. The training set is randomly divided for 6-fold LOOCV (Leave One Out Cross-Validation). Using abundance in RPKM of each contig as the only feature, AUC (Area Under Curve) on testing data is calculated. The contig with the largest AUC value is selected as the fingerprint marker of the current individual.

Thirdly, for the selected fingerprint, we set a step size of 0.001 to plot a ROC curve during one iteration of 6-fold LOOCV. The optimal threshold for each ROC curve is determined as the closest point to the perfect performance point ([*Sensitivity*, 1−*Specificity*] = [1, 0]). The optimal thresholds among 6-fold LOOCV are averaged as the optimal abundance threshold for the fingerprint of the current individual.

#### Module 4: An individual-identifying decision tree

The contig with optimal abundance threshold can separate its host from the other individuals as a fingerprint. However, it only works for separating one corresponding individual from other candidates. Therefore, a decision tree is designed to identify each individual combined with the fingerprints of all of the candidates.

A decision tree is a tree-like structure with branches that represent decision-making steps to implement classification and regression. Each branching node of a decision tree has a clear decision rule which is easy to read and interpret. Therefore, in this study, using the abundance of individual-specific fingerprints as decision rules in each node, a decision tree is designed to determine “the host of unknown samples in the candidate population” to implement individual identification. That is, a series of sub-decisions are usually made: “Does the sample to be identified belong to individual 1 with the individual1-specific fingerprint?”, if yes, the tree ends the decision, if not, the next decision is made, “Does the sample to be identified belong to individual 2 with the individual2-specific fingerprint?”…… until the leaf node in the decision tree is reached. Each node inside the decision tree is required to select an attribute, the abundance (RPKM) of a certain individual's fingerprint, to identify the individual as the corresponding host of the input skin microorganism sample.

The attribute order to be selected in the decision tree to identify the host in the candidate individuals is determined by Equation (5),


(5) $$\begin{array}{c} a^{*}=\underset{a\;\epsilon \;A}{\arg\min}\;Gini_{index}\left(D,a\right) \end{array}\;$$

and the attribute a^*^ means the individual that currently should be identified, where *A* is the candidate individuals (attributes) set. And *Gini*_*index*_(*D, a*) is defined in Equation (6),


(6) $$\begin{array}{l} \begin{array}{c} {Gini}_{index}\left(D,a\right)=\overset{V}{\underset{v=1}{\sum }}\frac{\left|D^{v}\right|}{\left|D\right|}Gini\left(D^{v}\right) \end{array}\; \end{array}$$

where ***V*** is the number of all decision nodes, and ***v*** is the ***v***^***th***^ decision node. *D*^*v*^ is the subset of *D* whose RPKM values are more than a threshold, *D* is the current sample set, and *Gini*(*D*^*v*^) is defined in Equation (7).


(7) $$Gini(D^{v})=\overset{\left|Y\right|}{\underset{k=1}{\sum^{\text{​}}}}\overset{}{\underset{k\prime \neq k}{\sum^{\text{​}}}}p_{k}pk\prime =1-\overset{\left|Y\right|}{\underset{k=1}{\sum^{\text{​}}}}p_{k}^{2}$$

And *p*_*k*_(*k* = 1, 2, …, |***Y***|) is the percentage of all samples of individual *k*, |***Y***| is the number of individuals in the dataset *D*^*v*^.

The smaller the *Gini*(*D*), the higher the purity. The individual that minimizes the Gini index is the optimal one for the following division.

After determining the individual to be decided, the decision tree uses an individual-specific fingerprint to perform a simple decision rule: “If the RPKM value of someone's fingerprint in the sample to be classified exceeds its threshold, the sample is assigned to that individual, otherwise it does not” to determine whether the unknown sample is from that individual.

Each branching point of the tree corresponds to each fingerprint, and the two branches represent whether the input sample has the abundance higher or lower than the threshold for the current fingerprint. The root node contains all individuals and each leaf node represents each individual. The construction of the decision tree was implemented in the Python *sklearn* package (Pedregosa et al., 2011). Metagenomic data from an unknown individual was aligned to the fingerprint contigs of all the candidates, and the corresponding contig abundances in PRKM were input into the decision tree. Using these individual-specific DNA microbial fingerprints and their corresponding thresholds as decision rules in branches of the tree, the unknown microbial skin sample is assigned to its host.

## Results

### Data description and preprocessing

In our experiment, we use the dataset from iHMP (Oh et al., 2014, 2016), composed of 616 metagenomic sequencing samples from skin microbial communities collected at 3 time points from 17 skin sites of 12 healthy individuals (hereinafter termed HV01-HV12). The 3 time points start at (T1). The second time point starts about 2 or 3 years later (T2) and a third about 5 weeks later after T2 (T3). Seventeen skin sites characterized physiologically as dry, moist, or sebaceous, include the following: antecubital fossa (Ac), alar crease (Al), back (Ba), cheek (Ch), external auditory canal (Ea), forehead (Fh), hypothenar palm (Hp), inguinal crease (Ic), interdigital web (Id), manubrium (Mb), occiput (Oc), popliteal fossa (Pc), plantar heel (Ph), retroauricular crease (Ra), toenail (Tn), toe web space (Tw), and volar forearm (Vf). Most symmetric sites were collected on the right side; only 52 were collected on the left. The metagenomic data were sequenced with 2 × 101bp pair-end reads by Illumina HiSeq. A detailed description can be found in Oh's studies (Oh et al., 2014, 2016).

**Figure 2:**
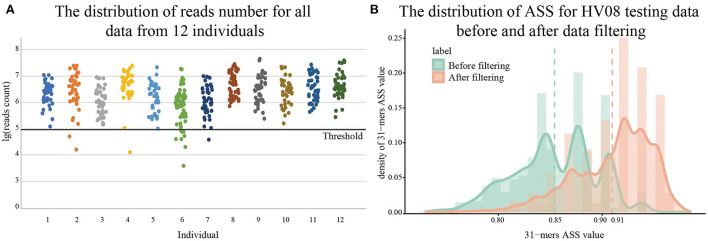
Preprocessing of metagenomic sequencing data. The scatterplot in **(A)** shows the distribution of read counts of samples from 12 healthy volunteers (HV). The data with the log of reads number fewer than 100,000 are removed. The bar chart in **(B)** shows the improvement of ASS values by data processing on the test dataset identifying HV08. The overall distribution of ASS values for 31-mers obtained after data processing can be seen as having shifted to the right in the figure, which means data processing improved the quality of *k*-mers we want to find. Dotted lines in the figure denote the mean value.

The scatter plot of the read number distribution for each sample is given in Figure 2A. Ten samples with extremely low sequencing depth, fewer than 100,000 reads in our case, were removed. Then 107 samples from three sites, plantar heel, toenail, and toe web space, were removed because their microbiomes are strongly related to living habits and affected by living environment and only shared a few markers with other body sites, according to a previous study (Schmedes et al., 2017). In total, 499 samples remained with which to perform the following analysis, and they are listed in Supplementary Table 1. Removing data of low sequencing depth helps us to identify high-quality individual-specific *k*-mers. Figure 2B shows the difference in ASS values of the detected individual-specific *k*-mers before and after data filtering for HV08. Average Sensitivity and Specificity (ASS) measures the discriminatory ability of each *k*-mer to separate the positive from the negative (see Methods). After filtering, the ASS value of individual-specific *k*-mers improved from 0.85 to 0.91 and is thus more convincing.

**Figure 3:**
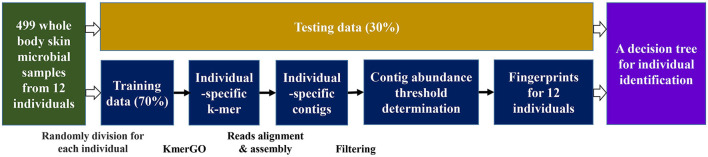
The flow chart shows the framework of our whole experiment. After data preprocessing, the samples of each individual are divided into the training set and the test set according to the 7:3 ratio. After a series of operations on the training data, we obtained fingerprints for 12 individuals and examined the testing data by a decision tree.

The experiment design and processing steps are shown in Figure 3. The 499 samples from 17 body sites of 12 individuals are divided into the training set and the testing set randomly with a 7:3 ratio. Based on the identified individual-specific *k*-mers by KmerGO (Wang et al., 2020), the individual-specific contigs are assembled based on reads containing the individual-specific *k*-mers. The contig with a determined abundance threshold is set as a fingerprint for each individual, and a decision tree is constructed for donor identification on the testing data.

The remaining 499 samples are randomly divided into a training set and a testing set with a ratio of 7:3 for each individual. Because of the imbalance between the size of negative and positive groups, the negative group is randomly under-sampled to the size of positive groups. The training set is further randomly divided for 6-fold LOOCV (Leave One Out Cross-Validation) for optimal parameter determination during the following experiments.

### Detection of individual-specific *k*-mers

The files passing data filtering were input to detect individual-specific *k*-mers. We set *k* = 31 in this study. Because the discriminatory ability varies for each individual, the ASS threshold is set between 0.8 and 0.9, as shown in Supplementary Table 2. Notably, there are 26,472 31-mers presents on all body sites for all individuals except for HV06, which might be called HV06-specifically absent 31-mers.

**Figure 4:**
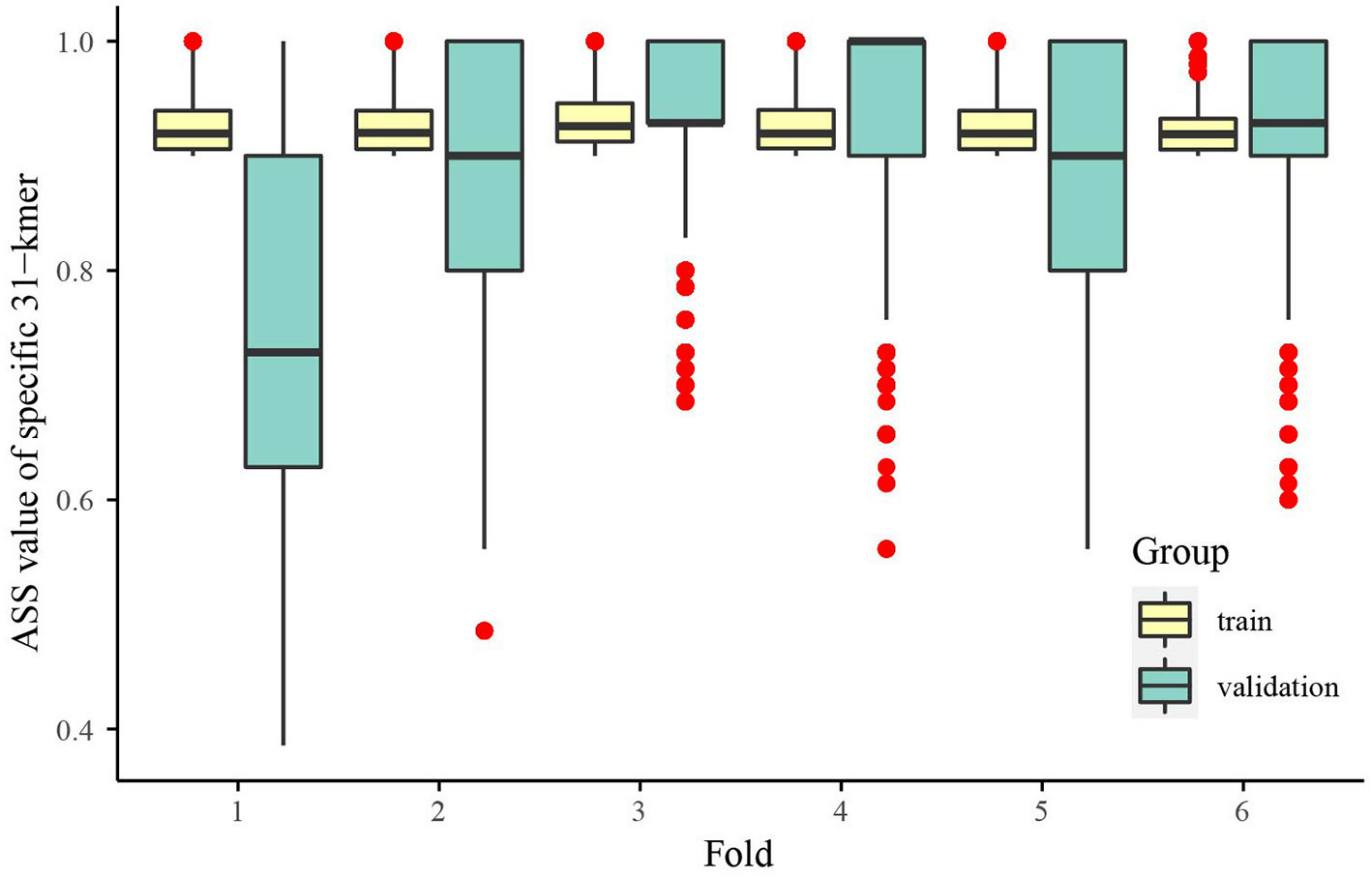
Boxplot of ASS value of HV08-specific 31-mers in 6-fold LOOCV. In each fold, even fold one, the *k*-mers obtained from training data maintain a high level (the average ASS is higher than 0.7, with most 0.85) of ASS in validation data, which means that these *k*-mers could effectively identify HV08.

As an example, HV08 has a total of 42 samples from 14 body sites collected at 3 time points, which were randomly divided into 30 training samples and 12 testing samples before the training process. The samples from HV08 are considered the positive group and the samples of other individuals are the negative group. Meanwhile, 64 negative samples were randomly selected from 320 training samples from the other 11 individuals. KmerGO (Wang et al., 2020), our previously developed group-specific *k*-mer detection tool, was adopted to identify the HV08-specific 31-mers. Taking the ASS threshold as 0.9, during the six round experiments of 6-fold LOOCV in the training set, there are 70,120, 31,084, 28,599, 23,823, 42,218, and 22,087 HV08-specific 31-mers detected respectively. This means that any one of these specific 31-mers could separate HV08 skin samples from the other individuals with averaged sensitivity and specificity higher than 0.9. When these specific 31-mers were evaluated in the validation sets, 33,721 (48.09%), 20,356 (65.49%), 25,585 (89.46%), 21,568 (90.53%), 24,340 (57.65%), and 21,422 (96.99%) 31-mers achieved ASS higher than 0.8, as shown in Figure 4. Among the 6 rounds of LOOCV, 5 out of 6 achieved an ASS median higher than 0.9, which means that the specific *k*-mers identified by KmerGO had sufficient discriminatory power to identify HV08 from the other individuals. Next, using ASS ≥ 0.9 as the threshold in the whole training set, we identified 29115 HV08-specific 31-mers. Among them, 26,911 (92.43%) 31-mers achieved ASS ≥ 0.8, and 10035 (34.47%) 31-mers achieved ASS ≥ 0.9 in the testing set, thereby illustrating the significant generalized performance of the identified individual-specific *k*-mers. The identification of specific *k*-mers for other individuals is given in Supplementary Table S2. The skin microbiomes of different individuals have different distinctiveness. HV08,09,10,11 found the most specific 31 mers with ASS ≥ 0.9. The number of individual-specific 31-mers varies among different individuals from 10^3^-10^4^. And 85% of specific 31-mers keep ASS ≥ 0.8 on the testing set for 9 out of 12 individuals.

Notably, each of the specific 31-mers has enough discriminatory power to separate the current individual from the whole candidate group with ASS higher than the set threshold instead of the combination of the identified 31-mers.

### Temporal stability of individual-specific *k*-mers

**Figure 5:**
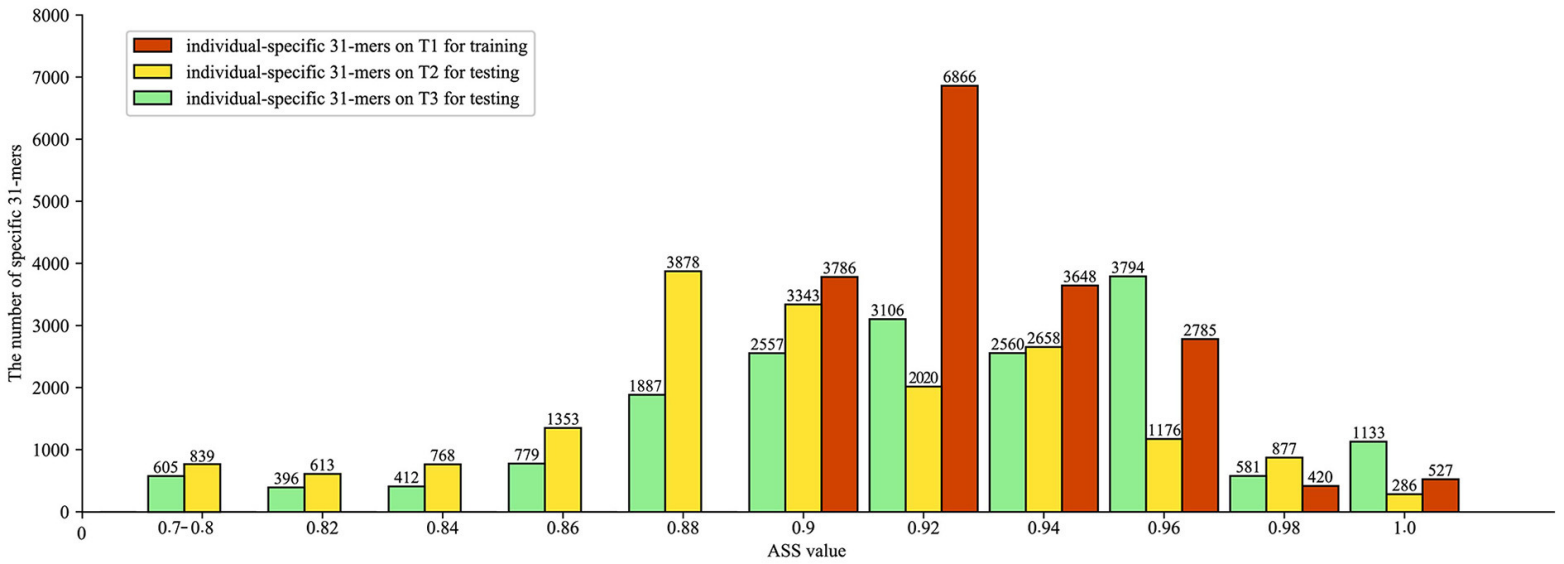
Evaluation of temporal stability of identified individual-specific *k*-mers, showing specific *k*-mers identified by ASS value of HV08 individual-specific 31-mers at three sampling times. Red bars represent individual-specific 31-mers on training, while yellow and green bars represent individual-specific 31-mers on two test datasets, respectively. The number above the bar chart represents the number of individual-specific 31-mers included in that ASS value.

Next, we assessed the temporal stability of individual-specific *k*-mers. Still using HV08 as our example, we detected HV08-specific 31-mers on the sample collected at T1 and tested it on samples collected at T2 and T3. As shown in Figure 5, the specific 31-mers detected on T1 exhibit ASS intensively distributed between 0.88 and 0.96. Specifically, among all 17,505 HV08-specific *k*-mers on T1, 16,952 (96.7%) achieved ASS ≥ 0.8 at T2, and 16282 (93.0%) achieved ASS ≥ 0.8 at T3, indicating that the individual-specific 31-mers have significant temporal stability.

For temporal stability, the other individuals exhibit very similar ASS tendencies in T2 and T3 with the testing set in Section Detection of individual-specific *k*-mers. As shown in Supplementary Table S2, the HV08, 09, 10, 11, and 12 keep high ASS around 0.9. And HV05 still shows the lowest temporal stability.

### Body site stability of individual-specific *k*-mers

Next, we assessed the body site stability of individual-specific *k*-mers. As we mentioned in the data description, there are three types of skin: dry, moist, or sebaceous, so we divided the data into two groups for training and testing by stratified sampling on the three different skin types, therefore, both the training set and testing set contain three skin types.

**Figure 6:**
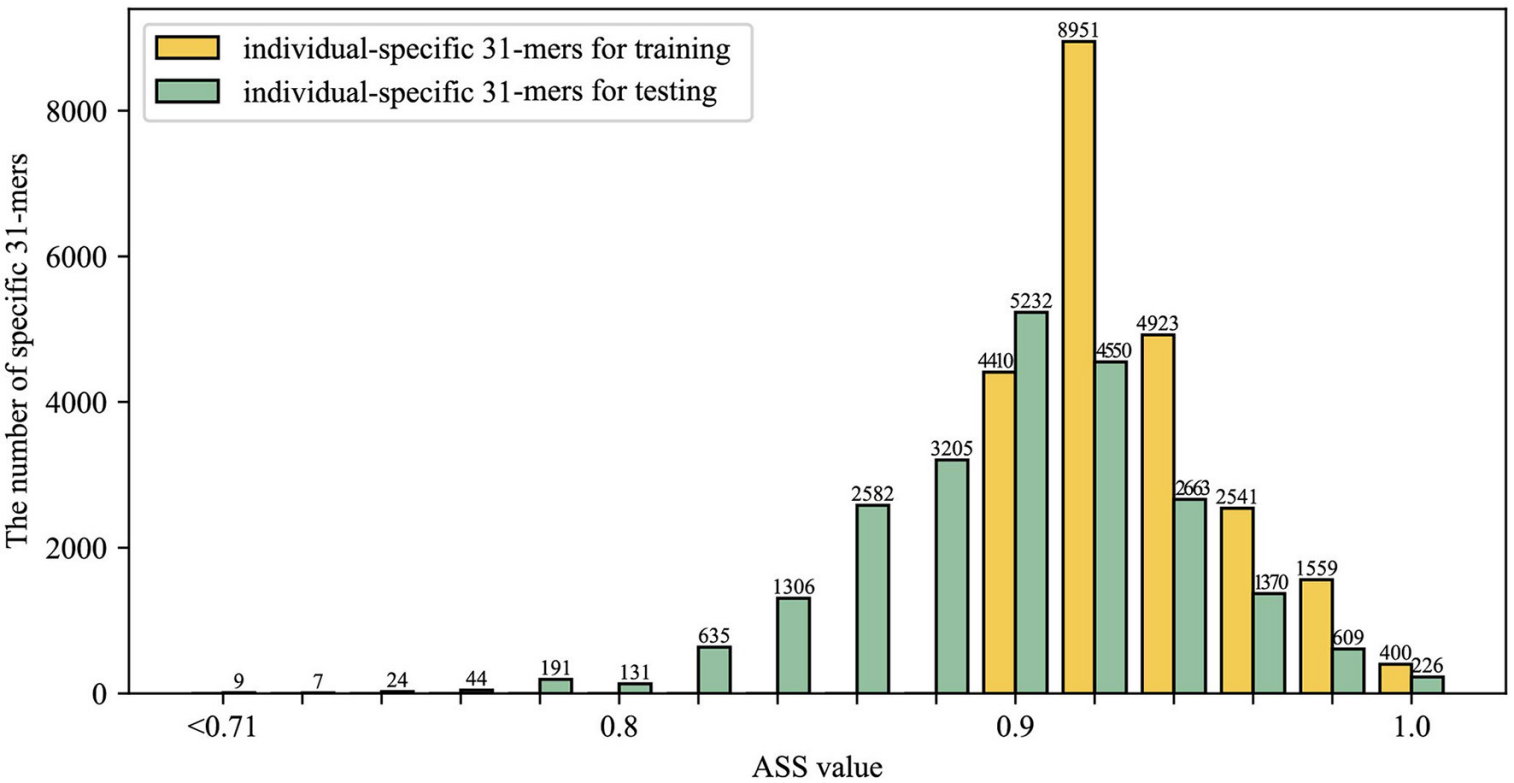
Evaluation of body site stability of identified individual-specific *k*-mers, showing specific *k*-mers identified by ASS value of HV08 individual-specific 31-mers on two groups samples. Yellow bars and green bars represent individual-specific 31-mers on training and testing, respectively. The number above the bar chart represents the number of individual-specific 31-mers included in that ASS value.

Still take HV08 as our example, we detected HV08-specific 31-mers on the sample from group one and tested it on samples from group two. As shown in Figure 6, there were 22,784 HV08-specific 31-mers detected on the training dataset with ASS higher than 0.9. On the testing dataset, 14,650 (43.2%) of them achieved ASS ≥ 0.9 and 22,509 (98.8%) achieved ASS ≥ 0.8, indicating that the individual-specific 31-mers have significant body site independence.

For body site stability, the other individuals exhibit very similar ASS tendencies on the testing set with the results on Section Detection of individual-specific *k*-mers, as shown in Supplementary Table S2. And HV08,09, 10, 11, and 12 keep high ASS and HV05 still shows the lowest ASS on the testing set.

### Individual-specific contigs as an individual's DNA microbial fingerprints

In Module 3, the identified individual-specific 31-mers were aligned back to the metagenomic sequencing reads, and reads that were perfectly aligned by more than two *k*-mers were kept for assembly. MEGAHIT (Li et al., 2015), a read assembly tool, was adopted to assemble these reads into individual-specific contigs. The number of individual-specific contigs ranged from 14 (HV06) to 22,299 (HV11), as shown in Supplementary Table 2. We found a significant variation in the number of individual-specific contigs among the 12 individuals. For example, HV08 had 29,115 individual-specific 31-mers assembled into 8,611 contigs with lengths from 203 to 33,760 bp, whereas HV05 had only 3,549 31-mers assembled into 51 contigs with lengths from 206 to 596 bp.

The contigs were aligned to reference genomes of the Bacteria database. Among all the genomes being aligned, we plotted a proportion-bar figure, as shown in Supplementary Figure S1. For 5 out of 12 hosts, all of the individual-specific contigs are only from *C. acnes*. And for the other 7 hosts, 50 to 90% of individual-specific contigs are from *C. acnes*, and the others are from *Lactobacillus crispatus* and *Siphoviridae*, etc.

**Figure 7:**
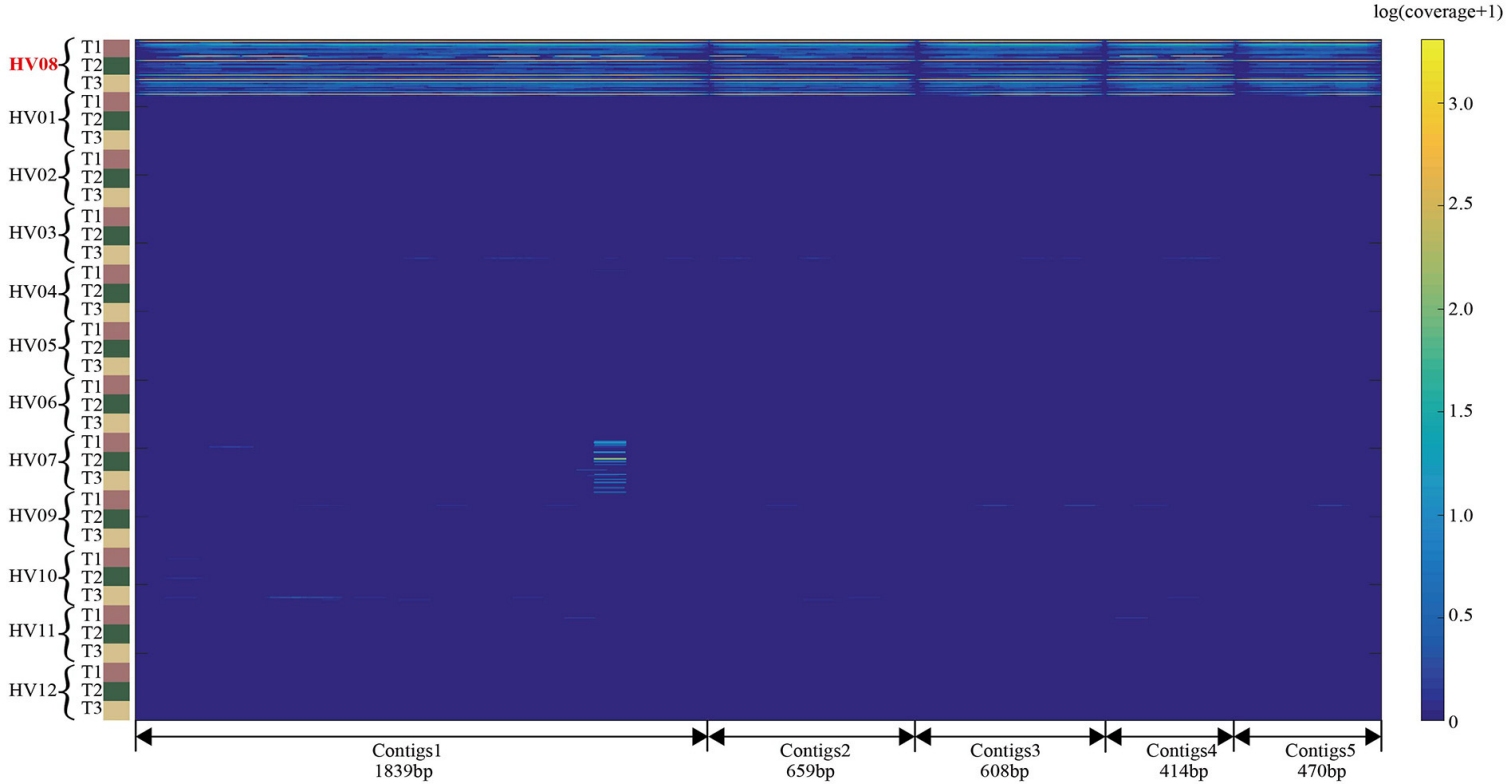
Coverage heatmap of the 5 HV08-specific contigs aligned by the 499 human skin samples from 12 healthy individuals. The coverage is the read-alignment depth in each nucleotide normalized by the number of million reads. To avoid the effect of a large span, we used the logarithm of (coverage+1) as the numerical value of the heatmaps. The horizontal axis is composed of each nucleotide of the 5 sequences, and the vertical axis is composed of each sample from each individual. The upper part of the heatmap is individual HV08, and the lower part is comprised of the other individuals.

Again, taking HV08 as our example, 8,611 HV08-specific contigs were assembled. The top 5 contigs with the highest RPKM in HV08 were aligned by all 499 metagenomic skin samples from the total 12 individuals, and the heatmap is shown in Figure 7. Each row is a metagenomic skin sample, which is composed of the coverage of each base in the contig. The logarithmetics coverage is represented by color. The blue indicates zero coverage and the yellow indicates high coverage. The lengths of these 5 contigs are 1,839, 659, 608, 414, and 470 bp, respectively. The coverage of the 5 contigs has the obvious difference between HV08 and the other 11 individuals, irrespective of time points and body sites. In a small 87 bp fragment in contig 1, the coverages of 11 out of 40 samples from HV07 show non-blue color, which is resulted in the ASS = 0.9 threshold. However, the length of contig 1 is 1839 bp, which is much longer than the 87 bp small fragment. Therefore, if contig 1 is taken as an HV08 fingerprint, the host still can be identified accurately.

### Biological characteristics of individual-specific *k*-mers and contigs

**Figure 8:**
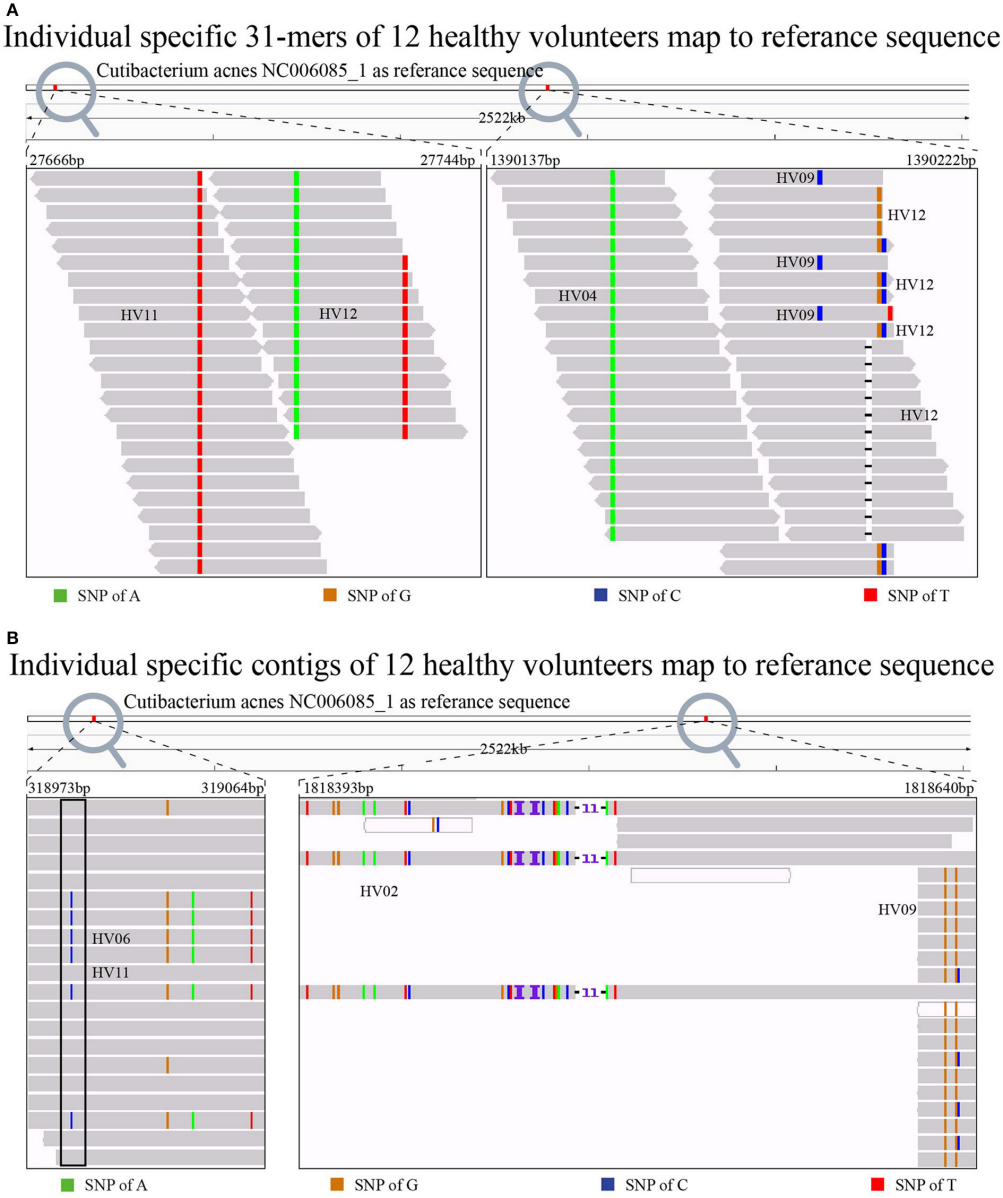
Individual-specific 31-mers **(A)** and contigs **(B)** alignments in *Cutibacterium acnes*, respectively. Each individual has his/her unique genetic variants, even though multiple individuals match the same region. For example, in the left of **(A)**, HV11 and HV12 both match the middle 318,681bp-319,906bp region, but HV11 has an individual-specific genetic variant of T, and HV12 has an individual-specific genetic variant of A. The same is found in the left of **(B)** where individual-specific contigs of HV06 and HV11 both align at position 318,983bp-319,104bp, but only HV06 has specific genetic variants at fixed position 318,989 with a base mutation of C.

Free from reference sequences and sequence alignment, the individual-specific *k*-mers are detected only on the metagenomic sequencing dataset. However, we were curious about the biological characteristics of the detected individual-specific *k*-mers and assembled contigs. Therefore, we aligned the individual-specific contigs to NCBI genomes (Coordinators, 2016) by BLAST (Altschul et al., 1990). Interestingly, we found that 55,343 (83.68%) of individual-specific contigs from the 12 individuals could be aligned to different regions of the *C. acnes* genome. This finding is consistent with the fact that the anaerobic type of *C. acnes* is most abundant on the skin in the whole body with distinct microbiota. Meanwhile, *C. acnes* manifests in individual-specific genetic variants in different people, and, as noted previously here, it has already been used as a feature in published work (Schmedes et al., 2017; Woerner et al., 2019). Moreover, we made a micromesh observation of contigs and *k*-mers alignments to *C. acnes* genomic reference sequence NC006285_1 with IGV (Robinson et al., 2011). We found highly consistent genetic variants over the whole body within individuals and distinct genetic variants between individuals. In Figure 8A, individual-specific 31-mers obtained from Module 2 are aligned to *C. acnes* with highly consistent genetic variants specific to HV04, 09, 11, and 12. Even when multiple individuals match the same sequence, it is possible to distinguish between them using their individual-specific genetic variants. Individuals HV09 and HV12 both match to 1,390,177bp−139,022bp, but HV12 has specific genetic variants at fixed positions 1,390,207 and 1,390,208 with a base mutation from T to G and from G to C, respectively, and a base deletion at position 1,390,205. HV09 has specific genetic variants at fixed position 1,390,196 with a base mutation from A to C. Figure 8B shows two genomic regions from the alignments of individual-specific contigs. The right subplot clearly shows that genetic variants of HV02 contigs are significantly different from those of HV09 contigs at 1,818,304–1,818,672bp. In the left subplot, some individual-specific contigs from both HV06 and HV11 are aligned to 318,681– 319,906bp, but HV06 has a specific genetic variant at position 318,989 with a base mutation from T to C, while HV11 does not.

### An identifier for the twelve individuals based on DNA microbial fingerprints

**Figure 9:**
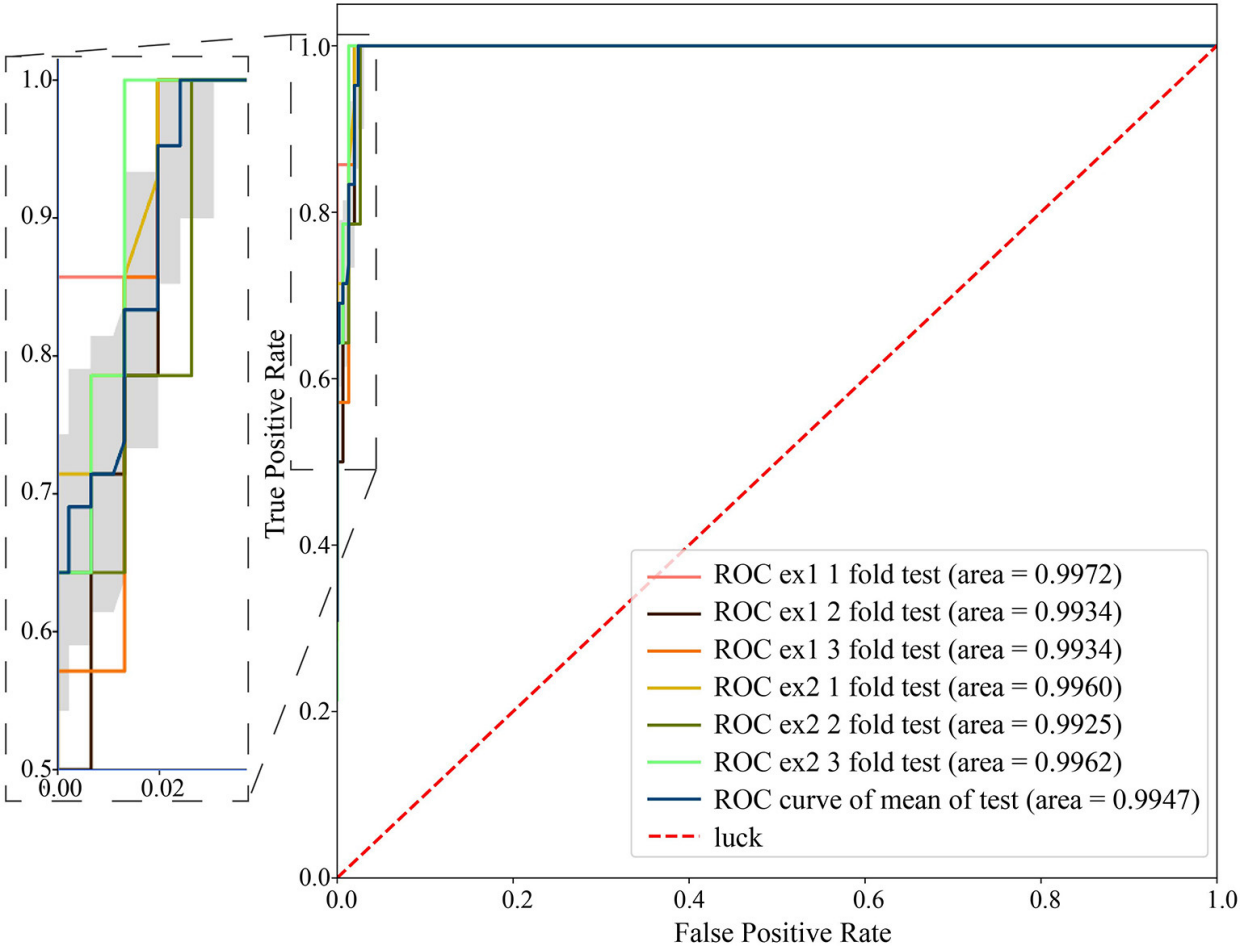
The ROC curve and AUC values were obtained from 6-fold cross-validation using the RPKM threshold of HV08's fingerprint. The classifier achieved a mean AUC value of 0.9947 on the validation data.

A fingerprint has greater utility if it works according to a simple rule, instead of a complex classifier combined with many features. Therefore, we used RPKM of an individual-specific contig as a fingerprint to identify individuals. For each individual, we selected an identified specific contig that could be aligned to a certain genomic region of *C. acnes* with proper length and abundance. The contig for each individual is given in Supplementary material. For fingerprint contig(s) for each individual, a proper threshold of contig abundance (RPKM) is determined by 6-fold LOOCV. Using HV12 as an example, a contig from *C. acnes* 1,373,635–1,373,596bp with one nucleotide insertion at position 1,373,624 is selected using abundance RPKM = 173 as the threshold. For an unlabeled sample, if the abundance RPKM of this contig is higher than 173, then the sample originates from HV12 with high probability. Still using HV08 as an example, the fingerprint is 23,786–25,608bp in *C. acnes* with 39 nucleotide variants. The threshold of HV08's fingerprint is determined as RPKM = 37 on the training set and validated with 6-fold LOOCV. The ROC curve and AUC values are shown in Figure 9. The mean value of AUC on validation data during the 6-fold LOOCV is 0.9947. The AUC, fingerprints, and thresholds of each individual are given in Supplementary Table 2.

Based on the RPKM threshold of each individual's fingerprint, we built a decision tree to identify each individual. The decision tree implements a branch judgment based on a simple decision rule, and each branch was decided by a threshold of one individual's fingerprint. For example, the samples from HV08 are only identified by the abundance of HV08's fingerprint, the contig from 23,786–25,608 bp in *C. acnes* with 39 nucleotide variants with RPKM greater than 37.

**Figure 10:**
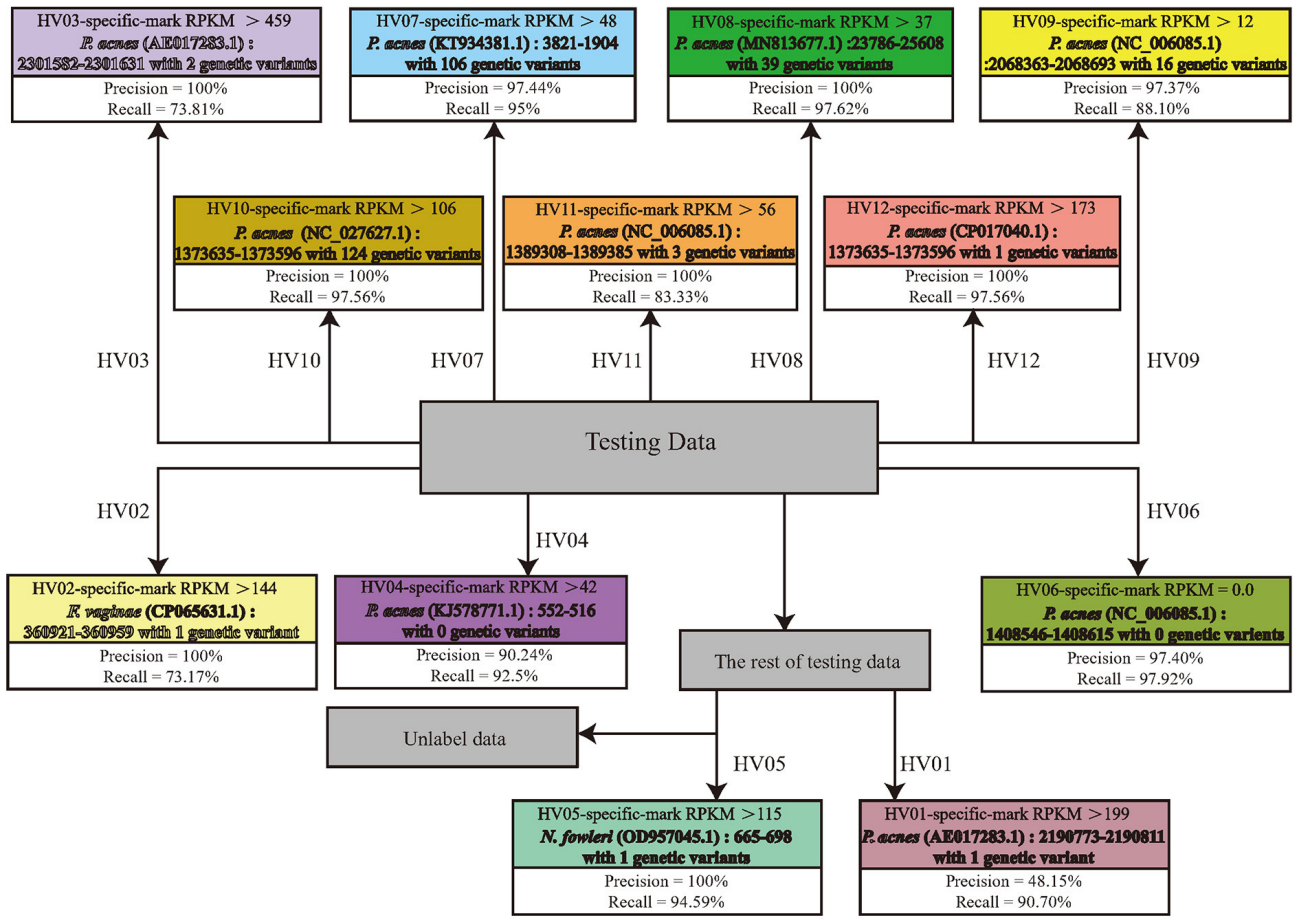
The graph shows the result of using microbial fingerprints as features to classify individuals with a decision tree. The dark gray node in the center of the decision tree is the root node, representing all unlabeled samples. The other colored leaf nodes represent all individuals.

The structure and the decision rule of the decision tree are shown in Figure 10. From the upper half of the tree, we observed that HV02, 03, 06, 07, 08, 09, 10, 11, and 12 are identified with high precision and recalled with a single decision rule, respectively. Other individuals are identified after two decision nodes. For example, the samples from HV01, 05 are identified with their fingerprint after HV02, 03, 04, 06, 07, 08, 09, 10, 11, and 12. The details of individual fingerprints are shown in the tree nodes, including the fingerprint's position and the number of genetic variants in the genome of *C. acnes*, the abundance threshold of the fingerprint, and the precision and recall.

The 149 testing metagenomic sequencing data from the whole-body skin of the 12 individuals were aligned to the 12 fingerprint contigs, and the corresponding contig abundances in PRKM were input into the decision tree. Using these individual-specific DNA microbial fingerprints and their corresponding thresholds as decision rules in branches of the tree, most of the unknown microbial skin samples are assigned to their host. Seven individuals achieved 100% precision with their fingerprints, and others achieved more than 90% precision. And the recall rate is between 73 and 97%. And 35 samples cannot be labeled in the decision tree.

**Figure 11:**
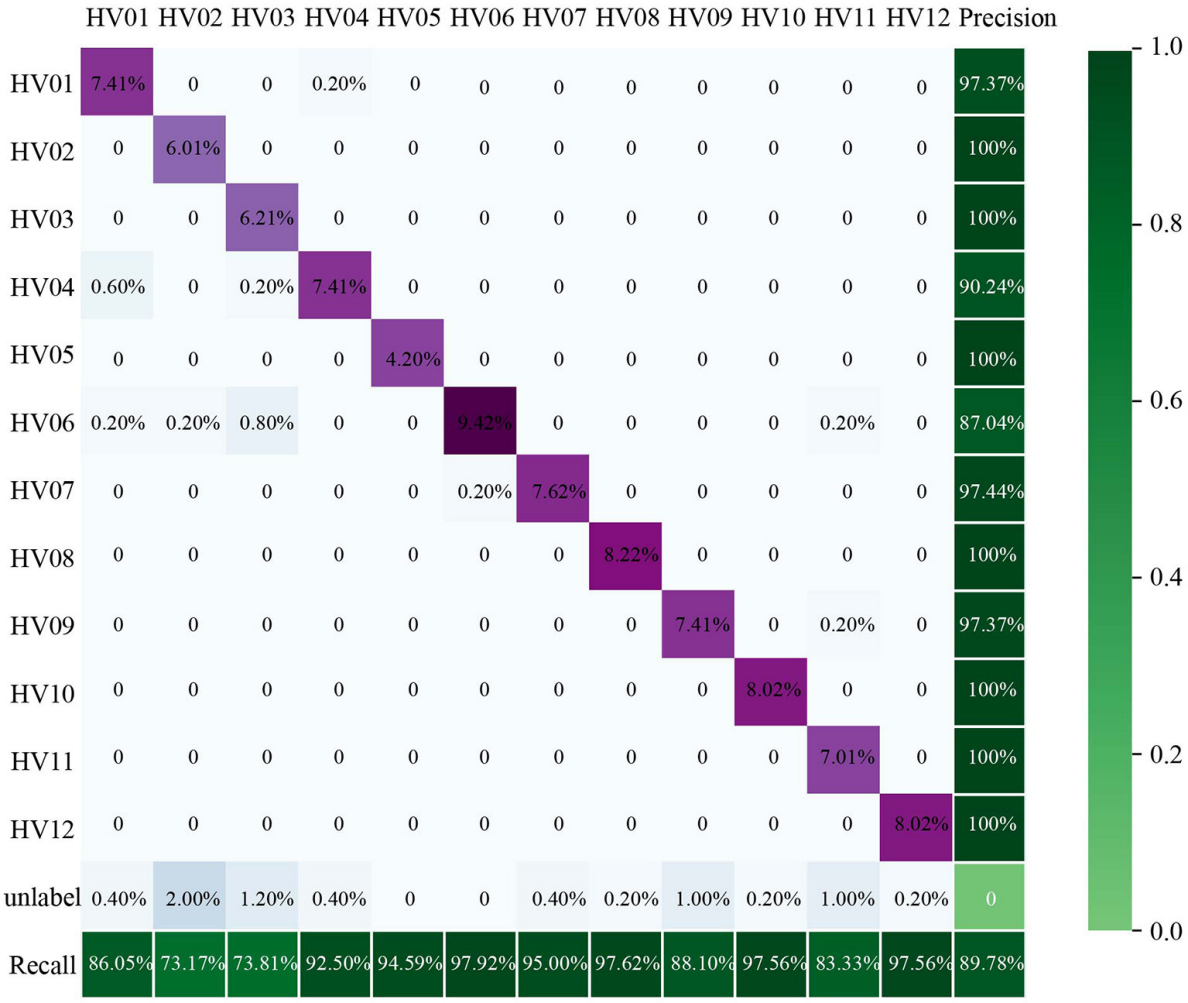
The confusion matrix shows the result of using microbial fingerprints as features to classify individuals with a decision tree. The row and column in the confusion matrix represent the label identified by the decision tree and the authentic label of samples, respectively. The sum of each column is the actual number of samples of this individual. The purple blocks on the diagonal represent the samples that have been correctly classified. The last row and the last column in the matrix are the recall and precision, respectively. Darker color correlates with higher accuracy of the classification.

Predicting precision and recall for each individual is shown as a confusion matrix in Figure 11. The row and column in the confusion matrix represent the identified label and the true label of the samples, respectively. The number in each block represents the number of samples, the true label of which is the corresponding column identified as the label of the corresponding row. The proportion of these samples out of all samples is shown below. The purple blocks on the diagonal are the samples that have been correctly classified to their donors. The green blocks in the last row and the last column are the corresponding recall and precision for each individual, respectively. As the color becomes darker, the performance of the classification increases. The precisions and recalls of HV07, 08, 10, and 12 are all higher than 90%. The precision of HV02, 03, 05, 08, 10, 11, and 12 is up to 100%.

To test how the model performs when the sample does not belong to any individuals in the training data, we downloaded 10 skin metagenomic sequencing data (listed in Supplementary Table S3) outside of the 12 individuals. And for 9 out of 10 samples, there are no reads that can map to any of the 12 fingerprints, so the samples are classified to the unlabelled node by the decision tree. And for only one sample, the reads map to the HV07's fingerprint with RPKM = 2.2, which is far smaller than the threshold of PRKM = 48, therefore, the sample is also classified as the unlabelled node.

## Discussion

Our study offers a novel skin microbial fingerprint identification framework. We verified the assumption that an individual's whole-body skin shares some consistent genetic variants. With the proposed framework, individual-specific, temporally stable, and body site-independent genetic variants in skin microorganisms were detected, which could be used as “fingerprints” to identify their donors.

Free from any reference sequences, the proposed model obtained temporally stable, body site-independent individual-specific fingerprints based only on metagenomic sequencing data. For the metagenomic sequencing samples from 17 skin sites at 3 time points of 12 healthy individuals' skin, we identified fingerprints for all 12 individuals. Furthermore, 10 out of 12 individual-specific fingerprints could be aligned to *C. acnes*. Using the abundance (RPKM) threshold of individual-specific contigs, 7 out of 12 individuals were identified with almost 100% accuracy, and the total accuracy was 89.78%.

Fingerprints with genetic variants offer a nucleotide-level resolution to understand the skin microbial community. Because the large-scale metagenomic sequencing dataset from multiple body site of a large population is not available, our study only made initial exploration limited to 12 individuals. Therefore, the dataset from a larger population is required to obtain a more convinced conclusion.

Furthermore, the high consistency of most genetic variants for each tested individual located in *C. acnes*, the large between-individual variability, and the high within-individual genetic consistency, even across different body sites, allow for identifying individual-specific skin microbial fingerprints, thus providing a valuable reference point for forensic scientists and skin biologists with potential applications in both forensic and biological contexts.

## Data availability statement

The original contributions presented in the study are included in the article/Supplementary material, further inquiries can be directed to the corresponding author/s.

## Author contributions

Conceptualization and investigation: YW, YZ, and JS. Methodology: JS, YZ, and QC. Software: QC. Validation: YZ, QC, and CD. Formal analysis, resources, and data curation: YZ and JS. Writing—original draft preparation: YZ. Writing—review and editing, visualization, and supervision: YW and JS. Project administration and funding acquisition: YW. All authors have read and agreed to the published version of the manuscript.

## Funding

This research was funded by the National Natural Science Foundation of China (62173282 and 31872564), the National Key Research and Development Program of China (2018YFD0901401), Fujian Provincial S&T Project (2019N0001 and 2017FJSCZY02), Open Fund of Engineering Research Center for Medical Data Mining and Application of Fujian Province (MDM2018002), and Natural Science Foundation of Fujian (2018J01097).

## Conflict of interest

The authors declare that the research was conducted in the absence of any commercial or financial relationships that could be construed as a potential conflict of interest.

## Publisher's note

All claims expressed in this article are solely those of the authors and do not necessarily represent those of their affiliated organizations, or those of the publisher, the editors and the reviewers. Any product that may be evaluated in this article, or claim that may be made by its manufacturer, is not guaranteed or endorsed by the publisher.
