# Supplementary material for: Identifying individual-specific microbial DNA fingerprints from skin microbiomes
Source: Front Microbiol. 2022 Oct 6;13:960043. doi: 10.3389/fmicb.2022.960043 (PMC9583911; doi:10.3389/fmicb.2022.960043)
Supplement: Supplementary file 1 [file Data_Sheet_1.ZIP › Supplementary Note.docx]

**Supplementary Note**

Identifying Individual-specific Microbial DNA Fingerprints from Skin Microbiomes

1. **Comprehensively experiment description for all of the 12 individuals**
   1. **Detection of individual-specific k-mers**

(1) HV01 has a total of totally 43 samples from 14 body sites collected at 3 time points, which were randomly divided into as 30 training samples and 13 testing samples before the training process. The samples from HV01 are considered the positive group and the samples of other individuals are randomly selected with 64 as the negative group. Taking the ASS threshold as 0.8, 14452 HV01-specific 31-mers were detected. When these specific 31-mers were evaluated in the testing sets, 10084 (69.78%) 31-mers achieved ASS higher than 0.8.

(2) HV02 has a total of totally 41 samples from 14 body sites collected at 3 time points, which were randomly divided into as 30 training samples and 11 testing samples before the training process. The samples from HV02 are considered the positive group and the samples of other individuals are randomly selected with 64 as the negative group. Taking the ASS threshold as 0.8, 16740 HV02-specific 31-mers were detected. When these specific 31-mers were evaluated in the testing sets, 9104 (54.38%) 31-mers achieved ASS higher than 0.8.

(3) HV03 has a total of totally 42 samples from 14 body sites collected at 3 time points, which were randomly divided into as 30 training samples and 12 testing samples before the training process. The samples from HV03 are considered the positive group and the samples of other individuals are randomly selected with 64 as the negative group. Taking the ASS threshold as 0.8, 2402 HV03-specific 31-mers were detected. When these specific 31-mers were evaluated in the testing sets, 1704 (70.94%) 31-mers achieved ASS higher than 0.8.

(4) HV04 has a total of totally 40 samples from 14 body sites collected at 3 time points, which were randomly divided into as 30 training samples and 10 testing samples before the training process. The samples from HV04 are considered the positive group and the samples of other individuals are randomly selected with 64 as the negative group. Taking the ASS threshold as 0.9, 4225 HV04-specific 31-mers were detected. When these specific 31-mers were evaluated in the testing sets, 3771 (89.25%) 31-mers achieved ASS higher than 0.8.

(5) HV05 has a total of totally 37 samples from 14 body sites collected at 3 time points, which were randomly divided into as 25 training samples and 12 testing samples before the training process. The samples from HV05 are considered the positive group and the samples of other individuals are randomly selected with 64 as the negative group. Taking the ASS threshold as 0.8, 3549 HV05-specific 31-mers were detected. When these specific 31-mers were evaluated in the testing sets, 1389 (39.14%) 31-mers achieved ASS higher than 0.8.

(6) HV06 has a total of totally 48 samples from 14 body sites collected at 3 time points, which were randomly divided into as 32 training samples and 16 testing samples before the training process. The samples from HV06 are considered the positive group and the samples of other individuals are randomly selected with 64 as the negative group. Taking the ASS threshold as 0.8, 26472 HV06-specific 31-mers were detected. When these specific 31-mers were evaluated in the testing sets, 18946 (71.57%) 31-mers achieved ASS higher than 0.8.

(7) HV07 has a total of totally 40 samples from 14 body sites collected at 3 time points, which were randomly divided into as 30 training samples and 10 testing samples before the training process. The samples from HV07 are considered the positive group and the samples of other individuals are randomly selected with 64 as the negative group. Taking the ASS threshold as 0.9, 1894 HV07-specific 31-mers were detected. When these specific 31-mers were evaluated in the testing sets, 1884 (99.5%) 31-mers achieved ASS higher than 0.8.

(8) HV08 has a total of totally 42 samples from 14 body sites collected at 3 time points, which were randomly divided into as 30 training samples and 12 testing samples before the training process. The samples from HV08 are considered the positive group and the samples of other individuals are randomly selected with 64 as the negative group. Taking the ASS threshold as 0.9, 29115 HV08-specific 31-mers were detected. When these specific 31-mers were evaluated in the testing sets, 26911 (92.43%) 31-mers achieved ASS higher than 0.8.

(9) HV09 has a total of totally 42 samples from 14 body sites collected at 3 time points, which were randomly divided into as 30 training samples and 12 testing samples before the training process. The samples from HV09 are considered the positive group and the samples of other individuals are randomly selected with 64 as the negative group. Taking the ASS threshold as 0.9, 17420 HV09-specific 31-mers were detected. When these specific 31-mers were evaluated in the testing sets, 15690 (90.07%) 31-mers achieved ASS higher than 0.8.

(10) HV10 has a total of totally 41 samples from 14 body sites collected at 3 time points, which were randomly divided into as 30 training samples and 11 testing samples before the training process. The samples from HV10 are considered the positive group and the samples of other individuals are randomly selected with 64 as the negative group. Taking the ASS threshold as 0.9, 19552 HV10-specific 31-mers were detected. When these specific 31-mers were evaluated in the testing sets, 17563 (89.83%) 31-mers achieved ASS higher than 0.8.

(11) HV11 has a total of totally 42 samples from 14 body sites collected at 3 time points, which were randomly divided into as 30 training samples and 12 testing samples before the training process. The samples from HV11 are considered the positive group and the samples of other individuals are randomly selected with 64 as the negative group. Taking the ASS threshold as 0.9, 37374 HV11-specific 31-mers were detected. When these specific 31-mers were evaluated in the testing sets, 35198 (94.18%) 31-mers achieved ASS higher than 0.8.

(12) HV12 has a total of totally 41 samples from 14 body sites collected at 3 time points, which were randomly divided into as 30 training samples and 11 testing samples before the training process. The samples from HV12 are considered the positive group and the samples of other individuals are randomly selected with 64 as the negative group. Taking the ASS threshold as 0.9, 7126 HV12-specific 31-mers were detected. When these specific 31-mers were evaluated in the testing sets, 6969 (97.8%) 31-mers achieved ASS higher than 0.8.

In summary, as shown in Table S2, the skin microbiomes of different individuals have different distinctiveness. HV08,09,10,11 found the most specific 31mers with ASS≥0.9. The number of individual-specific 31-mers varies among different individuals from 10^3^-10^4^, And 85% of specific 31-mers keeps ASS≥0.8 on the testing set for 9 out of 12 individuals. The fingerprints of HV04,05,06,07,08,09,10,11,12 are significantly distinct and therefore can separate the host from the others with high accuracy. Notably, HV06 does not possess HV06-specific 31mers which are present in HV06 but are absent from other individuals. However, there are 26,472 31-mers present on all body sites for all individuals except for HV06, which might be called HV06-specifically absent 31-mers.

1. **The source of the fingerprints**

The identified individual-31mers for each individual were assembled into contigs. The contigs were aligned to reference genomes of Bacteria database. Among all the genomes being aligned, we plotted a proportion-bar figure, as shown in Figure S1. For 5 out of 12 hosts, all of the individual-specific contigs from are only from *Cutibacterium acnes*. And for the other 7 hosts, 50% - 90% individual-specific contigs are from *Cutibacterium acnes*, and the others are from *Lactobacillus crispatus* and *Siphoviridae* etc.


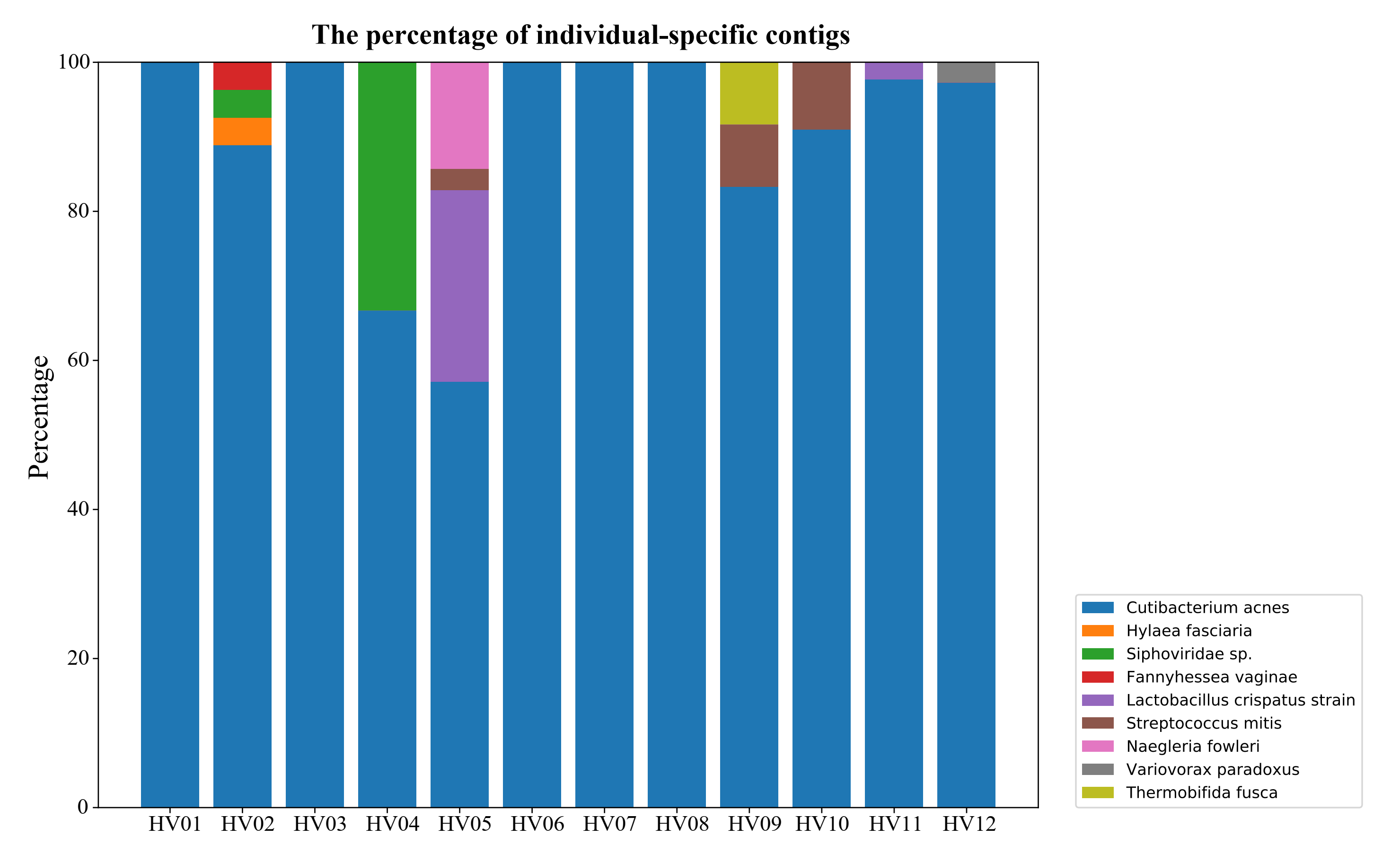


**Figure S1 The alignment distribution of individual-specific contigs**

1. **The classification results of SVM using fingerprint’s RPKM**

Decision tree is an easy “if-then” decision and keep the classifier in original feature space, which can give clear explanation of marker using. And it is still interesting to see the performance of the identified fingerprint in high-dimension nonlinear space. Therefore, we used same features to build SVM classifier using different kernels and grid searching for optimal parameters. And we found that the performance of RBF and linear does not have significant difference. And no improvement is observed compared with Decision Tree, which means that the features can implement good classification in original feature space, which reflect the effectiveness of the identified fingerprint from another side.

The confusion matrix and Roc curves are shown as follows:


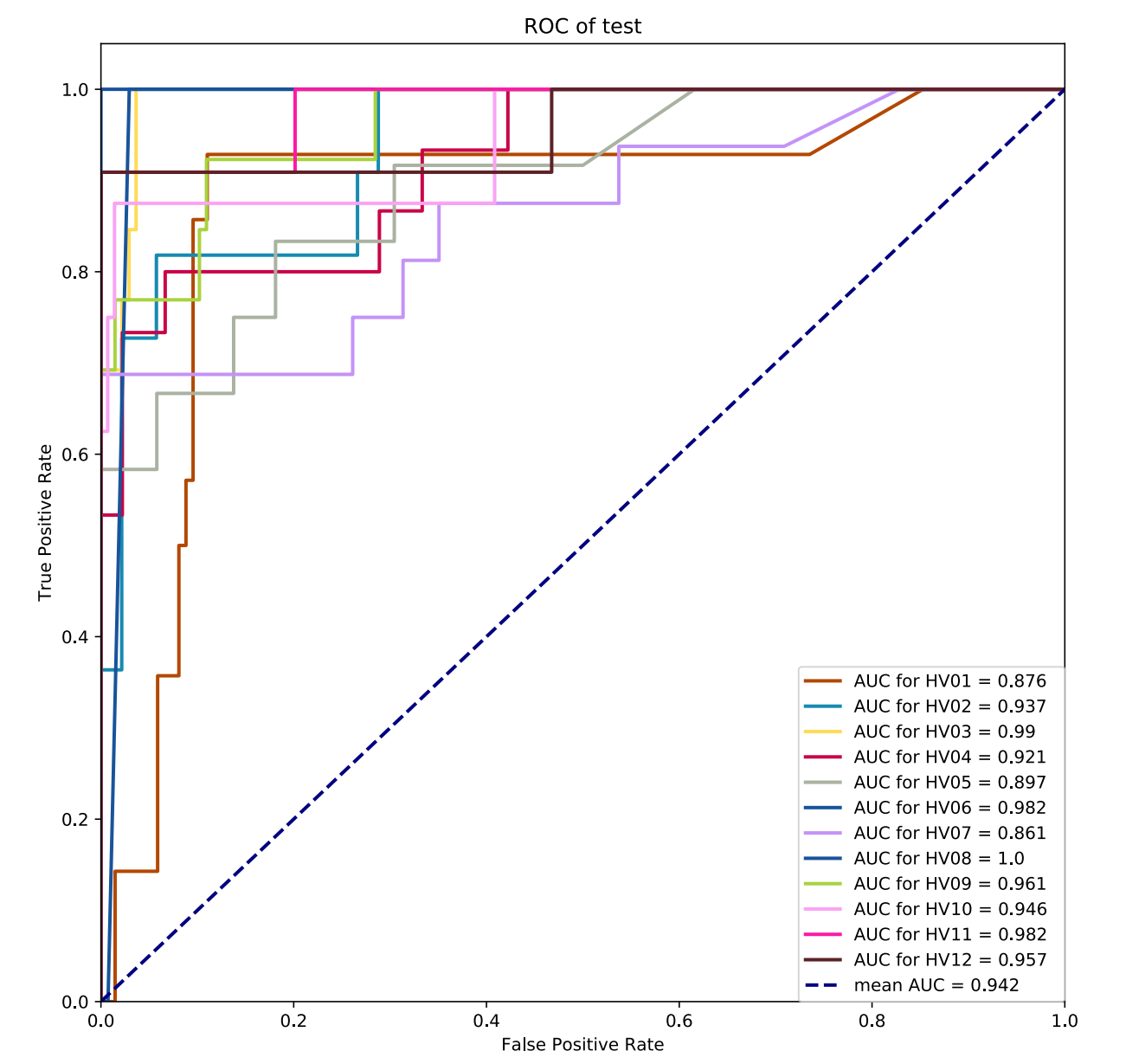


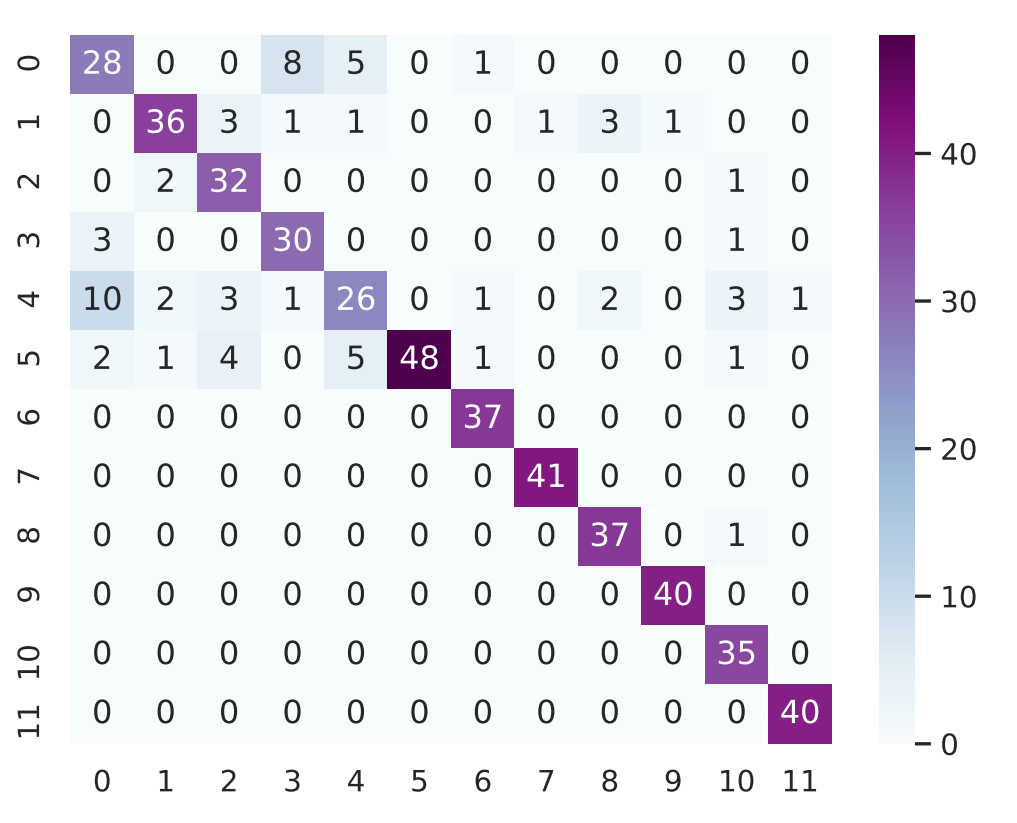


**Figure S2 ROC and Confusion matrix of SVM**
